# Instrumentation timing for manual K file and different pedatric rotary files systems in deciduous mandibular molars

**DOI:** 10.6026/973206300211429

**Published:** 2025-06-30

**Authors:** Vikram Jhamb, Ameer Akhil Ahmed Shaik, Priyanka Vinay Agrawal, Shamolie Shailesh Deshmukh, Indu Kumari, Harish Kumar, Hasina Khan, Pratik Surana

**Affiliations:** 1 Department of Pediatric and Preventive Dentistry, College of Dental Science, Amargadh, Gujarat, India; 2 Bachelor of Dental Surgery, Narayana Dental College, Nellore, India; 3 BDS, Jodhpur Dental College, Jodhpur, Rajasthan, India; 4 Intern, Bharati Vidyapeeth Deemed to be University's Dental College & Hospital, Pune, Maharashtra - 411043, India; 5 Department of Conservative Dentistry & Endodontics, Vananchal Dental College & Hospital, Garhwa, Jharkhand, India; 6 Department of Oral Pathology, Kalinga Institute of Dental Sciences, Bhubaneswar, Odisha - 751024, India; 7 Consultant Pediatric and Preventive Dentist, Hyderabad, Telangana, India; 8 Department of Pedodontics and Preventive Dentistry, Maitri College of Dentistry and Research Centre, Durg, Chhattisgarh, India

**Keywords:** Pulpectomy, rotary files, hand instrumentation, instrumentation time

## Abstract

Pulpectomy has been the standard treatment for necrotic primary teeth. However, hand files often results in longer chairside times. Pediatric rotary files made especially for primary teeth have been made possible by technological advancements, improving the effectiveness of pulpectomy. Therefore, it is of interest to compare instrumentation timing between Hand K files and pediatric rotary files (Prime Pedo^TM^ and Pro AF Baby Gold) in deciduous lower molars. Thirty deciduous molars in all were separated into three groups: Group I utilized Hand K-files, Group II employed Prime Pedo^TM^ and Group III utilized Pro AF Baby Gold. Examination revealed that the rotary and manual methods for treating deciduous teeth differed significantly in instrumentation time.

## Background:

The early loss of deciduous molars as a result of caries or other causes, like trauma, is the main issue in the field of pediatric dentistry [1]. This loss has detrimental effects on the dentofacial skeletal complex, influencing aspects such as soft tissue support, occlusion, proper development of the dentition and aesthetic appearance [2]. Furthermore, pulpal invasion is one of the most harmful effects of neglected dental decay in youngsters. Therefore, in cases of irreversible pulpitis, pulpectomy is considered as preferred treatment option [3, 44]. Despite being common, traditional hand instrumentation has dangers such as perforations in the canal, poor biomechanical preparation, instruments failure and lengthy treatment duration, all of which can have a detrimental behavioral impact on children. In contrast, rotary instrumentation has shown to enhance efficiency by requiring fewer appointments. As a result, more and more dentists are investigating the advantages of rotational endodontics in contemporary practice [5]. The use of NiTi rotary files for biomechanical preparation in deciduous teeth was first reviewed by Barr *et al.* who claimed that this technique was superior for eliminating the uneven walls of deciduous teeth [6]. In 2016, Introduction of a dedicated pediatric rotary system marked an important advancement in the pediatric endodontics field [7]. These pediatric rotary files are designed to be shorter in length, thereby facilitating procedures with young patients. Since that time, pediatric dental practisioners have employed a range of commercially available rotary files specifically tailored for pediatric use, including Kedo-S^TM^ (India), Pro AF Baby Gold^TM^ (India) and Prime Pedo^TM^ (India), Pedo flex (India), NT Pedo Gold (India) [8, 9-10]. Therefore, it is of interest to compare instrumentation timing between Hand K files and pediatric rotary files (Prime Pedo^TM^ and Pro AF Baby Gold) in deciduous lower molars.

## Materials and Methods:

The study included a total of 30 patients of age 4-8 years, who had deciduous mandibular molars designated for pulpectomy. To qualify, the teeth needed to exhibit one or more specific conditions while retaining at least two-thirds of the root: (a) pulpal necrosis, (b) signs of irreversible pulpitis, or (c) Furcal or periapical radiolucency. However, teeth were excluded if they presented any of the following problems: (a) pus discharge, (b) significant mobility, (c) cellulitis, (d) pulpal floor perforation, or (e) sinus. Moreover, patient who could not cooperate, had underlying health issues, or required medical considerations were also not included. The participants were divided into three groups using a random number table, and each primary tooth underwent a pulpectomy in a single appointment with local anesthesia. Subjective and objective measures of the anesthesia's efficacy were confirmed prior to starting further therapy. A rubber dam was employed to isolate the teeth throughout the procedures. After the initial removal of caries, an access cavity was prepared using BR 31, and the roof of this cavity was removed. The DG-16 Explorer was utilized to identify the starting orifice, followed by the application of no. 15 hand H files to establish the working length based on Ingle's radiographic method. Samples were divided randomly based on instrumentation used. In the first group (n=10), I carried out biomechanical preparation using hand ISO K files (21 mm, Mani INC, Japan) ranging from sizes 15 to 35, employing a quarter-turn pull-back technique. Up to size 30 K-files were used to prepare the mesial canals, while distal canals were managed with files reaching to size 35. In Group II (n=10), Prime Pedo^TM^ (Sky International Enterprises, India) rotary files were employed by sideward brushing motion at 300 rpm speed and 2.4 N/cm torque. A starter file was initially utilized for orifice enlargement, followed by P2 files for the wider canals and P1 files for the narrower ones. To prepare the apex, an endosonic file was also employed.

In Group III (n=10), instrumentation was performed by means of Pro AF Baby Gold (Dentobizz, India). Initial glide path was established via no. 15 and 20 K-files. For narrow canals, we utilized sequences B1 (4%, 20) and B2 (4%, 25) at 350 rpm and 1.5 N torque with continuous motion. For medium-sized canals, particularly the distal canals found in lower molars, sequences B2 (4%, 25) and B3 (6%, 25) were implemented. Irrigation was performed using sodium hypochlorite (1%) and saline in each group. Subsequently, the pulp cavity was dried using absorbent points and obturation was performed with Metapex (Meta biomed, Korea). The syringe was meticulously retracted from the canal following the insertion of the paste to the pulpal cavity. A moist cotton pellet was then positioned at the root canal orifice, with gentle compression applied to the Metapex. Following this step, chamber wall was cleaned to remove adhered Metapex by utilizing spoon excavator and post-obturation restoration was done with glass ionomer cement. Second appointment was made after 1 week for crown placement, as young patients often exhibit difficulty tolerating lengthy procedures and to maintain a standardized protocol. A digital stopwatch was utilized to assess instrumentation time. The timer was paused during saline irrigation and resumed upon the insertion of the first file. The operator documented the corresponding instrumentation time for each group. Data entry and analysis of instrumentation timings for experimental and control groups were conducted using Microsoft Excel. IBM SPSS Statistics (Version 22) performed data analysis, employing a one-way ANOVA to assess instrumentation time among three groups, followed by Tukey's HSD test for multiple comparisons.

## Results:

The time taken for instrumentation across all three groups was recorded in minutes, as shown in Table 1 (see PDF). For Group 1, which used K-files, the average time for canal instrumentation was 28.53 minutes. In contrast, Group 2, utilizing Prime Pedo files, averaged 15.24 minutes, while Group 3, using Pro-AF Baby files, had an average of 17.44 minutes. Further Tuckey post-hoc analysis revealed a statistically significant variance among group 1 and both rotary file groups (p<0.05), although there was no notable difference observed between the two rotary groups. Notably, quickest instrumentation time was achieved with Prime Pedo files, followed by Pro-AF files, while K-files required the furthermost time due to the manual technique.

## Discussion:

The discipline of dentistry is undergoing continuous transformation due to the integration of advanced technologies, thereby enabling dental practitioners to enhance the quality of care provided to their patients. Root canal preparation traditionally involves the use of hand instruments such as files, reamers, drills and rotary files. Nevertheless, methods reliant on handheld instrumentation often require considerable time and may lead to iatrogenic complications. In response to evolving trends, there has been a marked emphasis on increasing the efficiency and time-saving aspects of pulpectomy procedures. Significant advancements in rotary instrumentation have been in the domain of endodontics, particularly in relation to pediatric dentistry [11, 12]. Primary teeth are characterized by their ribbon-like shape and thin, elegantly curved roots, distinguishing them from permanent teeth. Prevailing rotary files designed for permanent teeth are not suitable due to specific design features [13, 14]. One significant drawback of utilizing these permanent rotary files in deciduous curved canals is the risk of lateral perforation [4]. As Kuo *et al.* suggest, the development of new rotary files tailored for primary teeth would be a more beneficial option [15]. During the current research, the instrumentation timing of the hand K-file was compared with two different pediatric rotary files. The Pro AF Baby Gold features five files made from NiTi-CM wire, which provides enhanced flexibility and resistance to cyclic fatigue, having constant taper of 4% and 6%, measuring 17 mm in total length, with an active length of 13 mm. These files are recommended for use in a brushing motion within well-lubricated canals [16, 17]. On the other hand, the Prime Pedo^TM^ pediatric rotary file system contains a starter file with an 8% taper and a length of 16 mm for orifice enlargement, along with P1 (#15, 6% taper, 18 mm) and P2 (#25, 6% taper, 18 mm) files are employed for preparing the coronal and middle thirds of the canal and an endosonic file with a 2% taper and a length of 18 mm for apical preparation, with its 2% taper facilitating a conservative approach to apex preparation [18, 19]. In our study, we selected an age range of 4 to 8 years to eliminate the possibility of root resorption and undeveloped roots [4]. Our study's results indicated a noteworthy and statistically significant decrease in biomechanical preparation time using both rotary systems. These results align with the research conducted by Panchal and coresearchers (2019) [20], which assessed K-files, H-files and rotary Kedo-S files. Several additional researches that reinforce these findings include the work of Crespo *et al.* (2008) [21], Jeevanandan *et al.* (2018) [22] and Makarem and coworkers (2014) [23]. Rotary systems have been shown to be effective in cleaning and shaping, leading to improved debris and tissue removal and reduced chairside time. However, in contrast, the research by Katge *et al.* (2016) indicates that using rotary two files results in longer instrumentation times compared to hand H-files involving primary molars [24]. While both rotary systems required less instrumentation time related to K-file group, the Prime Pedo file system showed shortest instrumentation time among other groups whereas no statistically significant variation was observed among the rotary file groups. The absence of a cross-over design is one of the current study's limitations, which would enable a comparison of all three groups within the same child-something not feasible due to sensitive sample selection. Future research could concentrate on analyzing segregated instrumentation times and performing inter-arch comparisons. It may also be beneficial to include other parameters such as obturation quality, pain perception and the cleaning efficiency of the files.

## Conclusion:

Pediatric rotary endodontic systems enhance convenience and reduce chairside time, making them suitable for children with behavioral issues. They serve as efficient substitutes for manual instrumentation and are widely regarded as the standard treatment for pulpectomies in primary teeth.
